# Two-way transcriptome approach for the identification of common gene targets across four insect orders and its validation in *Oxycarenus laetus*

**DOI:** 10.1038/s41598-025-08880-9

**Published:** 2025-07-09

**Authors:** Julie Rebecca Joseph Mathari, Habeeb Shaik Mohideen

**Affiliations:** Bioinformatics and Integrative Omics Laboratory, Department of Genetic Engineering, School of Bioengineering, College of Engineering and Technology, SRM Institute of Science and Technology, Kattankulathur, Chengalpattu, Tamil Nadu 603203 India

**Keywords:** Cotton, Insect, Transcriptomics, Bioinformatics, Broad-spectrum pesticide target, SDG12, Entomology, Genomics, Next-generation sequencing, RNA sequencing

## Abstract

Cotton production is negatively impacted by many insect pests from multiple orders, resulting in significant agronomic and economic losses. This study utilized a comparative transcriptomic methodology to discover conserved gene targets with potential applications in pest management across four insect orders that infest cotton: Hemiptera, Lepidoptera, Orthoptera, and Thysanoptera. A total of 104 publicly available RNA-Seq datasets, representing 17 pest species were de novo assembled in two ways, first was classified by read length (PE100 and PE150) and secondly as species-specific transcriptomes, and their qualities were assessed (alignment ≥ 90%, BUSCO ≥ 80%). Functional annotation utilizing insect-specific databases and orthology-based filtering identified three highly conserved genes, namely Arginine kinase (ArgK), Ryanodine receptor (RyR), and Serine/Threonine Protein phosphatase (STPP). These genes are involved in critical physiological functions, including ATP regeneration, calcium ion homeostasis, and phosphorylation-dependent signaling, and were enriched in pathways associated with insect development and stress response, including as JAK/STAT signaling and chitin metabolism. The study aimed to find broad-spectrum targets across several taxa; however, *Oxycarenus laetus*, a prominent sap-sucking pest of cotton, was chosen for downstream validation because of its increasing importance and ease of access for experimental research. The expression of ArgK, RyR, and STPP in *O. laetus* was validated by qPCR, affirming the biological significance of these targets and their functional conservation. This integrated methodology, which includes cross-order comparative transcriptome analysis and species-specific validation, illustrates a scalable approach for discovering essential molecular targets with translational potential in pest control. The results establish a basis for the development of RNAi-based or chemical strategies designed for cotton pest control.

**Supplementary Information:**

The online version contains supplementary material available at 10.1038/s41598-025-08880-9.

## Introduction

The pre-historic intertwined relationship between food, clothing, and shelter makes them indispensable for the survival of humans. The centuries-old practice of cotton cultivation extending from the Indus valley civilization to the modern age has been realized across continents and hence its sustained productivity has become crucial in diverse industries^1^. Cotton is referred to as white gold due to its economic importance. While its significance is undisputed, it faces a variety of biotic and abiotic stresses^2–4^. The influence of abiotic stresses such as high temperature, unfavorable rainfall and soil salinity cause a dramatic decrease in crop yield. Moreover, temperature anomalies and climatic zone displacement cause a change in the pathogen and pests’ distribution, which further impacts cotton yield^5^. Biotic stress caused by various arthropods poses a major hurdle in cotton production. Reports suggest that around 1300 pests affect cotton of which 40 are key pests^6,7^. Insect species recognized as major pests of cotton include *Agrotis* spp.^8^, *Bemisia tabaci*^9^, *Amrasca biguttula biguttula*^10^, and *Helicoverpa armigera*^11^. Notably, two mealybug species*, Phenacoccus solenopsis* (solenopsis mealybug)^12^ and *Maconellicoccus hirsutus* (pink hibiscus mealybug)^13^, have become increasingly prevalent. In addition, Hemipteran pests such as the southern green stink bug (*Nezara viridula*)^14^, brown marmorated stink bug (*Halyomorpha halys*)^15^, and *Thrips palmi*^16^ have emerged as persistent threats to the crop yield. Other economically important pests include *Pectinophora gossypiella* (pink bollworm)^17^ and *Oxycarenus laetus* (dusky cotton bug)^18^, both of which have demonstrated resistance to *Bacillus thuringiensis* (Bt) cotton varieties. According to recent reports from the Central Institute for Cotton Research (CICR)^19^, these pests collectively represent a substantial challenge to cotton productivity and integrated pest management practices in affected regions.

*O. laetus* has been documented as a multi-crop pest in Egypt, Sudan, Uganda, Congo, Kenya, Tanzania, Angola, and Malawi. *Oxycarenus* is a substantial genus with fifty-five species, of which six have been identified as pests of cotton. *O. laetus* significantly deteriorates and compromises the lint quality of cotton crops and consistently degrades the quality of bolls, thereby impacting the weight of cotton seeds and oil content, and thus rendering it a significant pest that requires immediate attention^20–23^.

Numerous strategies are employed for the management of these pests which include cultural, biological, behavioral, mechanical, chemical, and microbial control of pests^24^. The uncontrolled use of pesticides and their negative effect on the environment led to the development of Integrated Pest Management (IPM) in the late twentieth century. It recommends monitoring pest populations, identifying pests, and adopting eco-friendly pest-control techniques^25^. The emergence of transgenic crops such as Bt cotton led to the control of many pests. However, the Cry toxins exhibit little efficacy against sap-sucking and piercing insects^26,27^. There is a vital need to develop novel gene markers which may efficiently control a broad range of pests^28^.

Next-generation sequencing (NGS) techniques have increased the efficiency and decreased the cost of sequencing, allowing faster comparison of gene expression profiles and gene identification^29^. Early insect RNA Sequencing (RNA-Seq) studies often included basic annotation analysis to classify genes that were active in a specific tissue of a species^30^. With advancements in assembly, annotation, and expression quantification techniques, researchers now are more inclined to employ a comparative strategy to identify the transcriptional foundation of phenotypic variety. This may require comparing different species, sexes, tissue types, life stages, and other factors^31^. In recent years, the study of insect transcriptomes has been used to analyse gene expression profiles for biotechnological applications. A study that investigated the mechanism for thiamethoxam resistance in whiteflies revealed that overexpression of cytochrome P450 imparted resistance in this species^32^. Genes imparting resistance to pyrethroid, a widely used pesticide have been reported in *B. tabaci*^33^. Research found that phototransduction, cuticle alteration, and oogenesis/embryogenesis in pea aphids altered gene expression among reproductive morphs^34^. The silencing of the arginine kinase gene via RNA interference in *H. armigera* markedly affected energy metabolism, resulting in stunted growth, decreased pupation, and heightened mortality, indicating its viability as a molecular target for transcriptome-oriented pest management approaches^35^. A comparative midgut transcriptome analysis of Bt-susceptible and Bt-resistant Indian strains of pink bollworm (*P. gossypiella*) identified 1,741 differentially expressed unigenes, featuring down-regulation of established Bt resistance-associated genes (e.g., APN, ABCA, ABCG8, cadherin) and up-regulation of genes related to metabolic resistance (e.g., cytochrome P450, GST, carboxylesterase)^36^.

There exists a rising interest in identifying new pest targets to minimize non-specific responses in non-target species. Earlier studies have found genes that may be targeted in pests of the same genus^37^, as well as the same species but distinct developmental phases^38^. The present study aims; i) to perform a comparative cross-order transcriptome analysis to identify common genes that can be novel targets for the management of cotton pests, ii) to employ a two-way transcriptome approach to comprehensively capture gene diversity and consistency across four orders, and iii) to use *O. laetus* as a model organism to validate the targets identified.

## Materials and methods

### Data selection 

A comprehensive literature survey was conducted through the Centre Institute for Cotton Research (CICR) web portal (https://www.cicr.org.in/database/dbcottonpest.html) to identify major pests of cotton that led to the identification of thirty-seven primary pests. The NCBI SRA portal was browsed with scientific names of the pests to shortlist RNA-Seq data based on their availability. Filters such as “species name”, “paired-end sequencing”, “Illumina”, and “fastq” file format were employed and this resulted in 826 hits. Further filtering was done based on i) the read length, ii) adult stage, iii) untreated/control samples. The dataset selection was done to ensure uniform representation of insect orders was maintained and the details are listed in Table 2. A detailed list of the samples taken for the analysis is provided in the supplementary table S.T1. The datasets were downloaded in fastq.gz format using IBM Aspera CLI version 3.9.2.1426.c59787a^39^. The flowchart given in supplementary Fig. 1 illustrates the data selection procedure employed to narrow down the dataset.

### Quality check and processing 

The quality assessment of the samples was conducted using FastQC v0.11.9, developed by Simon Andrews in 2019 (http://www.bioinformatics.babraham.ac.uk/projects/fastqc/). The adapter sequences and other k-mers contained in the data were removed using Fastp version 0.20.1 tool which was used to automatically detect and clip the adapter sequences (Chen et al. 2018). Post trimming quality check was performed using FastQC v0.11.9 and the results were visualized using MultiQC^40^.

### Approach 1

#### Assembly and alignment

The cleaned reads were subjected to de novo assembly using Trinity version: v2.1.1^41^ to construct transcriptomes by two approaches. In the first approach, the RNA-Seq datasets of each species were processed independently as per their read length categories: 10 samples of PE100 and 14 samples of PE150. This yielded 24 species specific transcriptomes, corresponding to the different read length datasets.

All the transcriptomes were assessed for their overall alignment rate using bowtie2 version 2.4.2^42^ and the completeness of the assembled transcriptomes was evaluated against arthropoda-db10 using BUSCO version 5^43^, which estimates the completeness of the assembly based on the presence of universal single-copy orthologs.

#### Functional annotation and common gene identification

For the first approach, specialized insect-specific gene databases were searched for transcript annotations. Innate Immunity Genes in Insects (4IN) database (http://bf2i300.insa-lyon.fr:443/search/global), KONAGAbase^44^ and SWISS-PROT (Insecta)^45^ databases were chosen. These databases were downloaded to perform similarity search using Blast to identify genes matching with the assembled transcriptomic data.

### Approach 2

#### Assembly and alignment

In the second approach, species-specific transcriptomes were generated by merging data from both read length categories, resulting in a total of 17 transcriptomes representing each species under investigation. Data redundancy was eliminated utilising CD-HIT, and highly similar transcripts were aggregated into unigenes^46^.

All the transcriptomes were assessed for their overall alignment rate using bowtie2 version 2.4.2^42^ and the completeness of the assembled transcriptomes was evaluated against arthropoda-db10 using BUSCO version 5^43^.

#### Functional annotation and common gene identification

In the second approach, the databases mentioned in the approach 1 were merged to create a meta-database consisting of gene sequences from all the specialized databases. The database results were further filtered based on percentage identity and query coverage, which were set to 85% and 90% respectively. A meta-database was created, consisting of representative sequences for each gene in the individual databases.

A cross-species matrix to identify commonly expressed genes was prepared for both the approaches using R scripts. A combination matrix was created to understand the distribution of genes. 14 combinations were made which are: Hemiptera (H), Lepidoptera (L), Orthoptera (O) and Thysanoptera (T), HLOT, HLO, HLT, HOT, LOT, HL, LO, TH, HO, LT, and OT. Order- specific genes i.e., genes present only in specific orders were also analyzed. The common gene identification exercise was carried out using a dual method, which consisted of a database search being carried out against individual databases as well as the creation of a meta-database by merging the genes from all the databases. This was done to ensure unbiased identification of common genes irrespective of the approach used.

### Gene set enrichment analysis (GSEA) and KEGG pathway analysis

The common genes identified in the study were then subjected to KEGG^47^ enrichment analysis using the PathView^48^ package in clusterprofiler^49^ and GSEA analysis using an online server, Metascape^50^.

### Primer designing, RNA isolation, cDNA synthesis and qPCR

Gene-specific primers were designed for three genes using Primer3. The adult specimens of *O. laetus* were collected from the research fields of Tamil Nadu Agricultural University in Coimbatore. Total RNA was extracted using TRIzol reagent (Takara) and preserved at − 80 °C. The RNA concentration was measured using NanoDrop, and the cDNA library was constructed using the High-Capacity cDNA Reverse Transcription Kit (Applied Biosystems, Foster City, CA) under the following incubation parameters: 25 °C for 10 min, 37 °C for 120 min, and 85 °C for 5 min. The cDNA was used to optimize the PCR conditions to get maximum amplification of the products. The reverse transcription product was used for qPCR amplification on the QuantStudio 5 Real-Time PCR System, employing QuantStudio Design and Analysis Desktop Software v1.5.1 (Applied Biosystems), with primers specific to three target genes (refer to Table 1). The 10 μl RT-qPCR reaction mixture comprised 1 μl of cDNA, 5 μl of 2 × QuantiNova Probe PCR Master Mix (Qiagen), 5- 10 pmol of primers, and nuclease-free water to achieve the total volume. Relative quantification was conducted using the QuantStudio 5 Real-Time PCR System (with QuantStudio Design and Analysis Desktop Software v1.5.3, Applied Biosystems), comprising an initial denaturation of 10 min at 95 °C, succeeded by 40 cycles at 95 °C for 30 s and 55–60 °C for 1 min. All reactions were conducted with three biological replicates. qPCR data were analyzed using QuantStudio™ Design and Analysis Software v1.5.3 and melting curve analysis served as a measure of amplification specificity. Negative controls (absence of template) were employed to identify primer dimerization and nonspecific amplification.

**Table 1:** List of primers used in qPCR.

Gene	Trinity ID	Primer sequence
Arginine kinase (ArgK)	TRINITY_DN896_c0_g1_i1	Forward: GGTGGGCAGCAGACTTCTT Reverse: CTCCTTAAGAAGTACCTCACGAGG
Ryanodine receptor (RyR)	TRINITY_DN13058_c0_g1_i1	Forward: CGATTCTCTGAATTTGACTGGA Reverse: CTATGTTTGACCACCTTTCCTT
Serine/Threonine protein phosphatase (STPP)	TRINITY_DN58357_c0_g1_i1	Forward: CGATCGCAGTCAGGAATATCAG Reverse: TACGATGAATGTAAGCGGAGGTAT

## Results

### Data collection, assembly, and quality assessment

The SRA database was searched to retrieve datasets for the study and 104 RNA-Seq datasets were selected, comprising 41 Paired-End 100 (PE100) and 63 Paired-End 150 (PE150) datasets, representing seventeen species. Seven species in both the read lengths, seven exclusively in PE150 and three exclusively in PE100 were found. The detailed list of the samples taken for the study is mentioned in the supplementary table, S.T 1.

In the first approach, the application of Trinity v2.1.1 facilitated the creation of individual assemblies, yielding 24 transcriptomes tailored to specific read lengths. The evaluation of assembly quality was conducted through standard metrics, including total transcripts, contig N50, median contig length, and average contig length, as outlined in Tables 2 and 3. Among the PE100 assemblies, *T. palmi* exhibited the highest contig N50 of 4,845 bp, demonstrating remarkable assembly continuity. In contrast, hemipteran species such as *B. tabaci* and *M. hirsutus* recorded the highest transcript counts, suggesting intricate transcriptomes. The PE150 datasets consistently showed improved contig continuity and assembly metrics. For example, *T. palmi* achieved the highest N50 at 2,814 bp, while species like *A. gossypii* and *A. biguttula biguttula* exhibited significant transcript abundance and average contig lengths, highlighting the superior quality attained with longer read lengths.

**Table 2:** Data selection, Trinity, Bowtie2 and BUSCO stats for approach 1; PE100.

Name of species	Orders	Counts of transcripts		Stats based on all transcripts contigs			Stats based on only longest isoform per ‘gene’			Bowtie2	BUSCO (Arthropoda)
		Total Trinity ‘genes’	Total Trinity transcripts	Contig N50	Median contig length	Average contig	Contig N50	Median contig length	Average contig		
*Bemisia tabaci*	Hemiptera	1,116,033	1,483,570	763	381	599.88	627	356	529.56	92.35%	99.60%
*Phenococcus solenopsis*		41,617	54,769	2316	600	1215.4	1876	423	931.9	96.69%	97.30%
*Maconellicoccus hirsutus*		370,291	507,260	2778	409	1142.4	1079	325	645.43	95.51%	98.50%
*Oxycarenus laetus*		83,973	153,203	1903	446	963.61	1453	354	751.07	96.78%	99.70%
*Nezara viridula*		286,360	403,352	772	309	550.78	472	280	442.73	94.22%	94.60%
*Halyamorpha halys*		414,108	479,068	642	328	539.09	466	314	447.02	95.45%	98.30%
*Helicoverpa armigera*	Lepidoptera	82,136	126,835	2231	494	1104.1	1599	357	775.27	96.46%	98.60%
*Shistocerca gregaria*	Orthoptera	209,963	231,319	843	293	559.56	577	282	485.78	91.99%	94.90%
*Poecilocerus pictus*		137,233	163,463	1269	334	687.63	917	316	593.58	95.92%	95.80%
*Thrips palmi*	Thysanoptera	86,403	143,090	4845	528	1706.9	1691	351	795.46	97.58%	97.70%

**Table 3:** Data selection, Trinity, Bowtie2 and BUSCO stats for approach 1; PE150.

Name of species	Orders	Counts of transcripts		Stats based on all transcripts contigs			Stats based on only longest isoform per ‘gene’			Bowtie2	Busco (Arthropoda)
		Total Trinity ‘genes’	Total Trinity transcripts	Contig N50	Median contig length	Average contig	Contig N50	Median contig length	Average contig		
*Amrasca biguttula*	Hemiptera	305,131	392,625	816	329	586.78	528	304	482.61	93.98%	95.20%
*Aphis gossypii*		149,552	188,712	2160	480	1042.9	939	397	678.94	95.12%	98.80%
*Bemisia tabaci*		518,860	622,582	737	361	593.22	544	342	501.77	92.58%	98.20%
*Dysdercus cingulatus*		63,586	72,857	874	361	609.31	698	343	545.25	92.02%	69.80%
*Phenacoccus solenopsis*		214,723	279,186	2188	417	967.69	737	356	600.26	90.64%	97.00%
*Nezara viridula*		140,015	167,094	1426	378	766.06	734	347	587.06	89.74%	97.10%
*Halyomorpha halys*		307,284	371,122	916	337	620.59	512	314	479.39	94.06%	97.60%
*Empoasca onukii*		326,751	524,216	838	376	619.28	576	322	504.11	90.11%	96.00%
*Spodoptera litura*	Lepidoptera	126,290	154,017	2182	425	988.83	1048	370	686.78	98.57%	97.90%
*Helicoverpa armigera*		175,104	255,621	1395	394	786.92	755	326	581.18	93.47%	95.10%
*Pectinophora gossypiella*		49,584	59,514	1364	481	844.23	1095	434	732.38	94.05%	84.60%
*Agrotis ipsilon*		40,962	55,164	1109	477	758.98	901	418	658.83	93.44%	66.40%
*Poecilocerus pictus*	Orthoptera	184,726	208,236	657	308	530.44	524	289	476.16	91.51%	83.00%
*Thrips palmi*	Thysanoptera	139,456	194,972	2814	468	1136.7	1028	384	714.65	94.46%	98.70%

The distribution of the data across 17 species is detailed in Table 4. In the table, cells designated as NA indicate that data was not available for that species for the specified read length. The evaluation of assembly quality for the merged species-specific transcriptomes (Table 4) involved analyzing bowtie2 alignment rates and BUSCO completeness scores, in addition to core assembly statistics including total Trinity genes, transcripts, and contig metrics. *B. tabaci* demonstrated the highest transcript count of 1,356,748 and a Trinity gene number of 1,030,515, underscoring its intricate transcriptome profile. *T. palmi* exhibited the highest contig N50 at 3,102 bp and the longest average contig length of 1,110.72 bp, reflecting exceptional assembly continuity. Hemipterans *P. solenopsis* and *M. hirsutus* demonstrated impressive assembly quality, achieving N50 values of 1,876 bp and 2,356 bp, respectively, along with BUSCO scores of 97.3% and 98.5%. Most of the species attained bowtie2 alignment rates exceeding 93%, with *O. laetus* and *P. solenopsis* achieving rates of 96.78% and 96.69%, respectively. Furthermore, *O. laetus* demonstrated robust contig integrity, achieving an average contig length of 826.62 bp, even with a moderate transcript count. The data highlights reliability and thoroughness of the combined assemblies among various insect species, offering robust transcriptomes for subsequent comparative and functional studies.

**Table 4:** Data selection, Trinity, Bowtie2 and BUSCO stats for approach 2.

Species name	Order name	Sample named as	Number of samples (PE100)	Number of samples (PE150)	Counts of transcripts		Stats based on all transcripts contigs			Stats based on only longest isoform per ‘gene’			Bowtie2	BUSCO
					Total Trinity ‘genes’	Total Trinity transcripts	Contig N50	Median contig length	Average contig	Contig N50	Median contig length	Average contig		
*Bemisia tabaci*	Hemiptera	BTS	11	6	1,030,515	1,356,748	721	386	589.26	732	400	599.87	92.35%	99.60%
*Phenacoccus solenopsis*		PSS	1	6	190,073	237,944	1876	411	893.71	940	368	667.42	96.69%	97.30%
*Maconellicoccus hirsutus*		MHS	4	NA	348,402	428,540	2356	372	966.41	1117	324	650.17	95.51%	98.50%
*Oxycarenus hyalinipennis/laetus*		OLS	4	NA	81,668	110,927	1595	393	826.62	1469	360	760.03	96.78%	99.70%
*Nezara viridula*		NVS	3	6	295,589	382,023	736	320	557.27	632	320	528.24	94.22%	94.60%
*Halyomorpha halys*		HHS	1	6	429,655	595,696	622	330	532.04	593	343	524.94	95.45%	98.30%
*Dysdercus cingulatus*		DCS	NA	1	55,032	59,096	826	358	593.28	744	347	561.76	92.02%	99.80%
*Aphis gossypii*		AGS	NA	6	135,249	159,829	1824	449	929.26	973	394	687.66	95.12%	98.80%
*Amrasca biguttula biguttula*		ABS	NA	6	214,473	248,628	792	357	600.77	688	337	559.25	93.98%	95.20%
*Empoasca onukii*		ES	NA	4	319,679	399,871	688	346	554.24	583	325	508	90.11%	96.00%
*Helicoverpa armigera*	Lepidoptera	HAS	4	6	143,044	218,736	1529	395	817.92	1545	390	807.74	96.46%	98.60%
*Pectinophora gossypiella*		PGS	NA	2	43,155	48,151	1277	478	817.38	1178	453	770.14	84.05%	84.60%
*Spodoptera litura*		SLS	NA	6	99,528	125,352	2293	426	1003.73	1392	373	765.31	98.57%	97.90%
*Agrotis ipsilon*	Orthoptera	AS	NA	1	40,710	48,977	972	448	697.48	900	419	658.9	93.44%	86.40%
*Shistocerca gregaria*		SGS	2	NA	176,170	192,578	987	310	609.36	696	297	541.13	91.99%	94.90%
*Poecilocerus pictus*		PPS	5	1	203,853	297,273	730	301	548.33	789	330	584.38	95.92%	95.80%
*Thrips palmi*	Thysanoptera	TPS	6	6	163,244	246,190	3102	425	1110.72	1434	388	810.1	97.58%	97.70%

### Identification of common genes by first approach

#### 4IN database

The information about the databases is mentioned in the supplementary table S.T 2. The 4IN database, encompassing genes associated with innate immunity in insects, produced a significant array of both unique and common protein targets among the four insect orders examined viz. Hemiptera, Lepidoptera, Orthoptera, and Thysanoptera. A total of 437 immune-related genes were identified within the assembled transcriptomes, with 118 considered potentially targetable as per the literature. The identified genes were linked to crucial immune pathways, such as the production of antimicrobial peptides (AMP), Toll and IMD signaling, the JAK/STAT cascade, MAPK-JNK pathways, and responses mediated by reactive oxygen species (ROS).

**Fig. 1:**
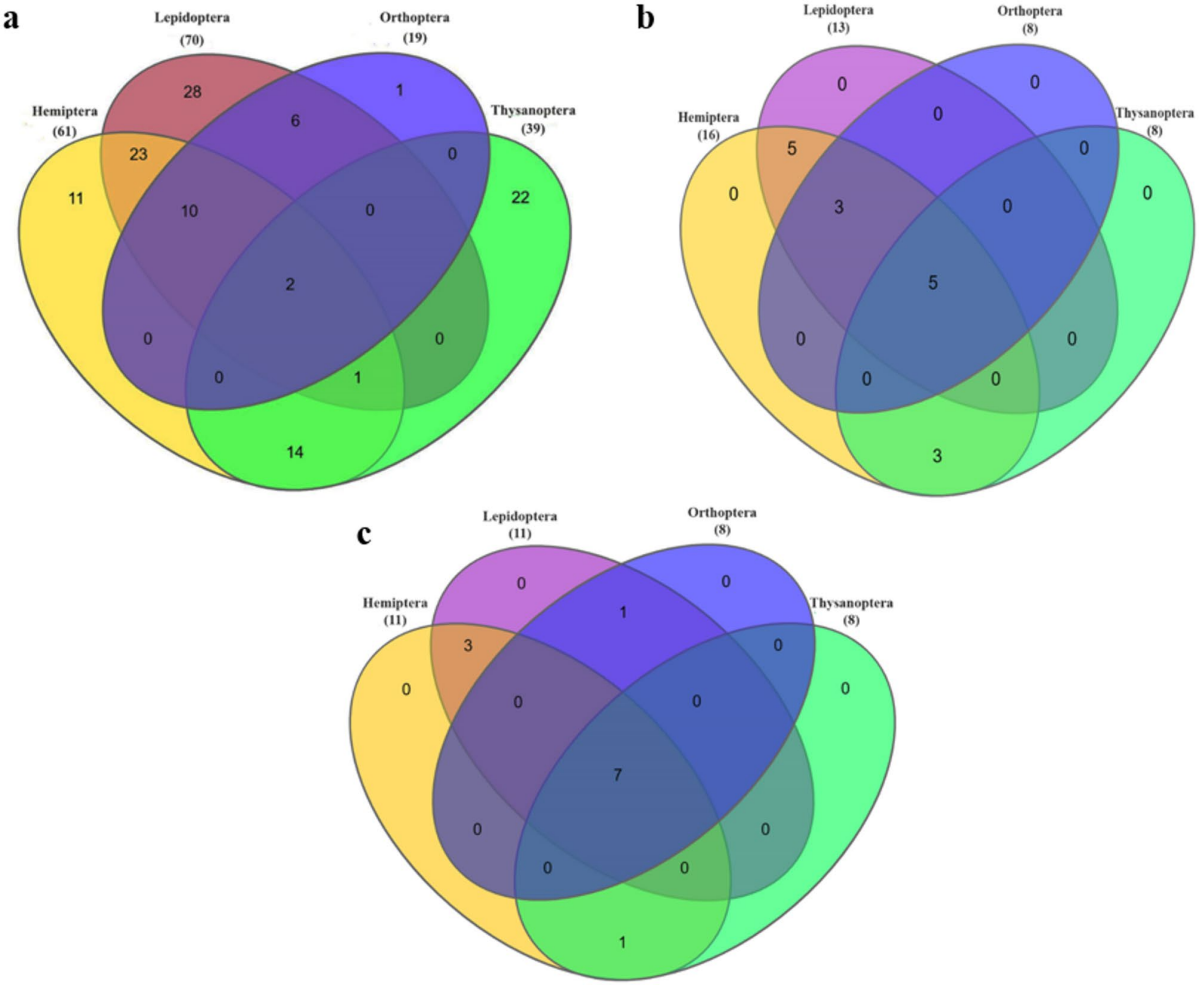
The Venn diagrams illustrate the distribution of targets among four insect orders: Hemiptera, Lepidoptera, Orthoptera, and Thysanoptera. The diagram in (**a**) represents results from 4IN database; (**b**) represents results from SWISS-PROT database; (**c**) KONAGAbase. Each section of the diagrams is color-coded to represent the different insect orders.

In the PE100 assemblies, eight genes linked to ROS signaling, the JNK pathway, and microbial recognition were identified as common across three species, suggesting a degree of conservation in immune response components. Furthermore, a total of 82 genes associated with cellular immune responses, encompassing both Toll and IMD pathways, were identified as being common to two species. Conversely, the PE150 datasets revealed a wider range of conservation: one gene related to the MAPK-JNK pathway was found in nine species; two genes associated with the JNK pathway were present in seven species; four genes within the same pathway were identified in six species; and five genes connected to the IMD pathway were shared across five species. Gene Px006614, which plays a role in the MAPK-JNK-p38 pathway, has been recognized as a conserved target. Additionally, a total of eight genes associated with autophagy were identified across three species, while 27 genes linked to the IMD pathway were detected in two species. The analysis of gene distribution revealed that Lepidoptera possessed the most (70) number of unique immune-related genes. This was followed by Hemiptera with 61, Thysanoptera with 39, and Orthoptera with 19. Only two genes were preserved across all four orders (combination HLOT), underscoring the evolutionary divergence of immune mechanisms. Nevertheless, multiple combinations demonstrated significant overlap for example, Hemiptera-Lepidoptera (HL) shared 36 genes, while Lepidoptera-Orthoptera (LO) shared 18, indicating a degree of conservation among these orders (Table 5; Fig. 1a). The genes associated with the Toll and IMD pathways were notably significant, highlighting their crucial functions in the regulation of antimicrobial peptides through NF-κB-dependent transcriptional cascades. Furthermore, genes associated with JAK/STAT signaling and ROS responses were identified. A significant discovery was the occurrence of C-type lectins, a category of pattern recognition receptors, across seven species from all four orders positioning them as promising universal targets. Additional potential targets encompassed serine/threonine kinases and serine proteases, both of which play a functional role in immune signaling and pathogen defense. Supplementary table S.T 3 provides a comprehensive overview of the distribution of these genes across 17 species. The findings illustrate the extensive capabilities of the 4IN database in documenting order-specific and partially conserved immune genes, emphasizing the promise of immune-related targets like C-type lectin, JNK-pathway regulators, and serine kinases for the advancement of cross-order pest management strategies.

**Table 5:** Summary of the database search across the different combinations of orders.

Combinations	4IN	Insecta-specific SWISS-PROT	KONAGAbase	Meta-database (before refinement)
HLOT	2	5	7	1815
HLO	12	8	6	24
HOT	2	5	7	29
LOT	2	5	7	None
HL	36	13	10	6
HO	12	8	8	17
HT	17	12	9	9
LO	18	8	9	None
LT	3	9	8	None
OT	2	5	7	None
Hemiptera	61	16	11	8
Lepidoptera	70	13	11	1
Orthoptera	19	8	8	None
Thysanoptera	39	8	8	None

#### Insecta-specific SWISS-PROT

The findings from the 4IN database present a broader and more immune-focused perspective, whereas the SWISS-PROT (Insecta) database offers a more refined set of conserved targets. This distinction highlights the curated nature of SWISS-PROT and its focus on experimentally validated proteins. The similarity search conducted on SWISS-PROT, focusing on the Insecta class, yielded 672 hits, with sixteen genes identified as targetable according to existing literature and functional annotation. Many of the remaining hits were identified as structural or housekeeping proteins and were therefore excluded from further analysis. A total of 336 genes (PE100) and 591 genes (PE150) were recognized as common among two or more species. Upon the exclusion of structural and housekeeping genes, the curated datasets exhibited consistent trends. In the case of PE100, there were 33 genes common across ten species, 30 genes shared among six species, and 89 genes found between two species. In PE150, a total of twelve genes were identified as common among 14 species, 33 genes among 13 species, and 101 genes shared between two species. Among the targetable genes, five were consistently identified across all 14 order combinations, prominently featuring serine/threonine protein phosphatases (STPP) and the ryanodine receptor (RyR) are two essential regulators of phosphorylation and calcium signaling, respectively. Three additional genes, comprising a STPP isoform and arginine kinase (ArgK), were identified as common across three insect orders: Hemiptera, Lepidoptera, and Orthoptera. A distinct group of four genes, comprising chitin deacetylase 1 and several STPP isomers, were discovered across six combinations that included Hemiptera, Lepidoptera, and Thysanoptera. There were sixteen targets in Hemiptera, thirteen in Lepidoptera, and just eight each in Orthoptera and Thysanoptera (Fig. 1b; Table 5). The overall cross-matrix results are provided in supplementary table S.T 5.

Most of the conserved targets were either common to two orders or specific to one, with a significant overlap observed between Hemiptera and Lepidoptera, which both contained five shared protein targets. Notably, five genes were identified as fully conserved across all four insect orders, underscoring their extensive evolutionary conservation and possible application as universal targets for pest control. The data indicates a notable decrease in the number of conserved genes as the range of taxa expands, yet it still underscores the presence of a strong core gene set that remains functionally conserved among cotton pest species. The results underscore the importance of the SWISS-PROT database in identifying stable, biologically relevant targets that are appropriate for comprehensive pest control approaches.

#### KONAGAbase

The examination of the KONAGAbase database, which includes genomic information from *Plutella xylostella* (diamondback moth), resulted in the identification of a fundamental group of insect gene targets that are preserved across various agricultural pest orders. Out of 2,407 genes mapped to the species in this study, eleven protein targets were consistently found in both Hemiptera and Lepidoptera, whereas eight targets were identified in each of Orthoptera and Thysanoptera. This variation indicates evolutionary divergence and potential functional specialization among these insect groups.

Significantly, seven protein targets were identified as conserved across all four insect orders (HLOT), indicating the highest degree of target conservation observed in this analysis. These targets hold significant potential for the advancement of comprehensive pest management approaches. Figure 1c demonstrates that the shared presence of these targets across different taxonomic orders highlights their evolutionary stability and crucial biological functions, indicating a significant potential for cross-order applicability in biocontrol strategies. From the identified pool of genes, twelve genes were further classified as targetable based on functional annotation and literature. These encompass well-defined targets including chitin synthase 1, chitin deacetylase, serine/threonine protein kinase, and acetylcholinesterase, the latter of which is among the most extensively utilized enzymatic targets in chemical pest management.

It is important to highlight that RyR and ArgK were included among the twelve targetable genes. RyR was found across all four orders and all 14 experimental gene-expression combinations, highlighting its widespread presence and strengthening its potential as a target for broad-spectrum biopesticides. In a similar way, ArgK, which has been previously validated by our experimental group, showed a consistent presence throughout the datasets, and corresponds with earlier findings that confirm its effectiveness in biocontrol. The analysis of gene frequency from the dataset indicated that 14 genes, such as RyR and ArgK, reached the highest combination score of 14, demonstrating their widespread presence across all tested conditions. This reinforces their categorization as essential genes and increases their appeal as targets for pest management approaches, owing to their consistency across various environments and taxa. An analysis of gene presence by order reveals that Hemiptera and Lepidoptera exhibit a greater number of targets with KONAGAbase (eleven each), in contrast to Orthoptera and Thysanoptera (eight each). This indicates a potential closer genomic alignment or shared ecological adaptations between Hemiptera and Lepidoptera. The overall cross-matrix results for KONAGAbase are provided in supplementary table S.T 4.

### Identification of common genes by second approach

**Fig. 2:**
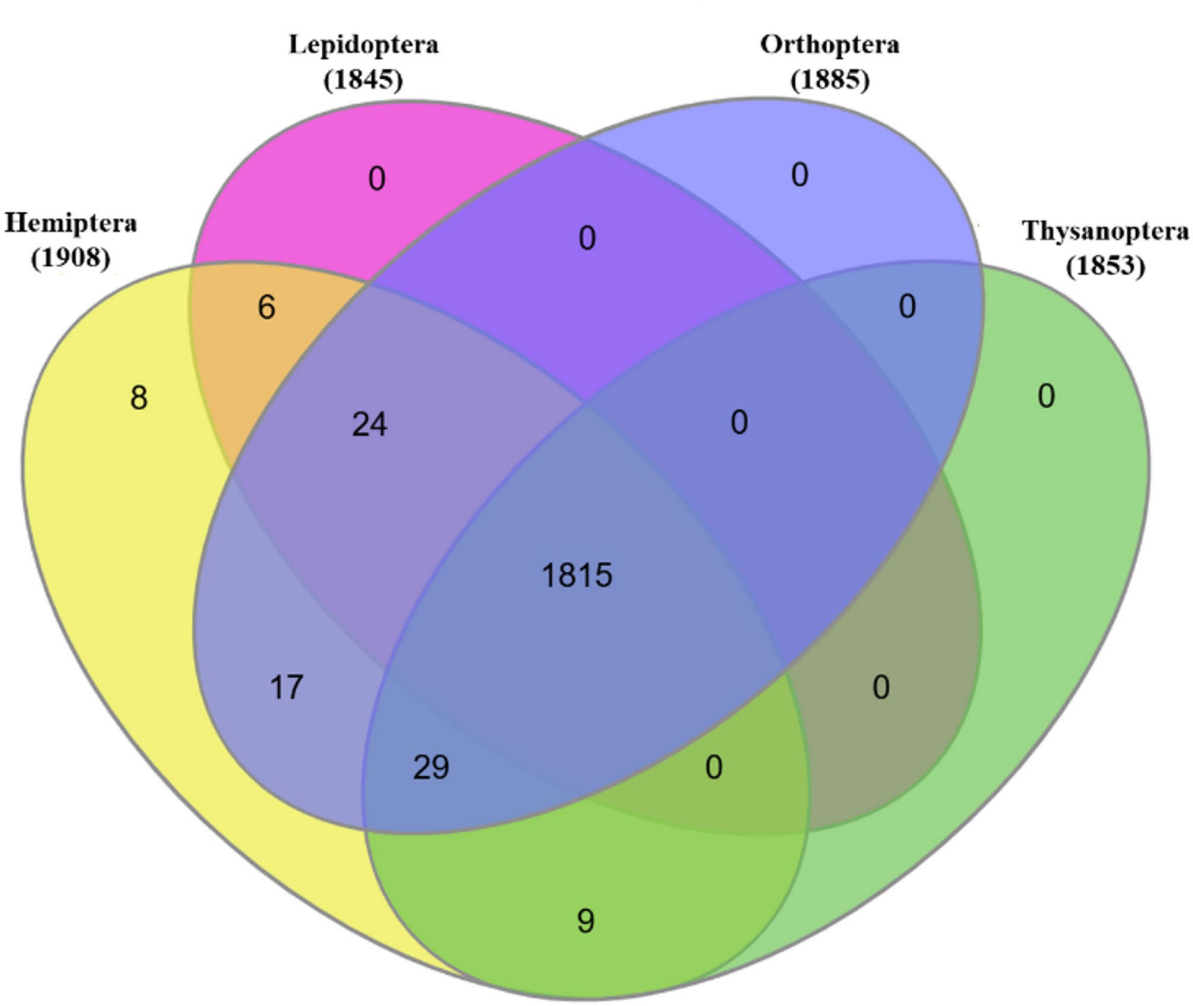
The Venn diagram illustrate the distribution of genes identified by the second approach among four insect orders: Hemiptera, Lepidoptera, Orthoptera, and Thysanoptera. Each section of the diagrams is color-coded to represent the different insect orders.

The integration of gene data collected from various databases resulted in the establishment of a meta-database that encompasses a total of 52,35,976 sequences. After conducting sequence similarity searches, a total of 48,733 results were selected for additional analysis. To enhance these findings, genes were meticulously filtered according to their established or predicted functional roles and by the potential they demonstrate for targeted pest control applications. A comprehensive multi-step filtering process revealed a total of 1,909 genes that may serve as potential molecular targets. The order-wise distribution of these genes is depicted in the Table 5. Venn diagram in Fig. 2 illustrates the distribution and frequency of these gene families across representative orders.

A total of 1,302 genes were classified into ten unique gene families, highlighting essential physiological and biochemical pathways in insects. The acetylcholine receptor family stood out with 615 gene entries, followed by chitin deacetylase with 263, STPP at 233, and ArgK with 174. Additional significant families comprised ecdysone receptor (9), vitellogenin (4), juvenile hormone-related genes (2), Ras-related genes (2), and odorant receptor (1). RyR, recognized as a significant target for biopesticides, was represented by three separate gene entries. Table 4 offers a detailed species-level representation. A comprehensive list of target genes, encompassing isoforms and relevant annotations, can be found in supplementary table S.T 6.

It is noteworthy that 1,302 genes were identified as conserved across all four orders, indicating the existence of a highly stable and potentially cross-order gene pool. Multiple smaller overlaps were identified, such as Hemiptera and Thysanoptera (29 genes), Hemiptera and Orthoptera (17 genes), Lepidoptera and Orthoptera (24 genes), and Hemiptera and Lepidoptera (6 genes). Interestingly, Hemiptera was the only order to display uniquely represented genes, totalling eight, which suggests potential lineage-specific adaptations or gene loss in the other orders.

The significant degree of overlap, especially the seven core targets present across all four orders underscores the importance of the above approach in identifying conserved, biologically essential genes. From the twelve targetable genes identified in this core set, the RyR and ArgK stood out as particularly significant. RyR was identified in every order and in all 14 expression combinations evaluated, highlighting its extensive significance. ArgK, validated through our previous experimental work, reinforces its potential as a strong candidate for cross-species biocontrol strategies. The findings collectively illustrate a methodical and effective strategy for determining conserved gene targets across various insect taxa, presenting robust candidates for the advancement of next-generation, species-wide pest management approaches.

### Summary from both the approaches

This work utilized two transcriptome assembly strategies: read length-based assemblies (Approach 1) and species-specific merging assemblies (Approach 2). Approach 1 entailed the analysis of 104 RNA-Seq datasets using Trinity v2.1.1, resulting in 24 transcriptomes categorized by read length were 41 PE100 and 63 PE150. PE150 datasets typically produced more extensive assemblies, as seen by elevated N50 values and average contig lengths in species such *as T. palmi* and *A. biguttula biguttula*. Simultaneously, Approach 2 employed Trinity to compile 17 species-specific transcriptomes by integrating reads from identical species, with the objective of achieving a more comprehensive transcript representation. Quality assessment via bowtie2 indicated alignment rates surpassing 93% for most species, while BUSCO analyses validated elevated completeness levels, with species such as *P. solenopsis* and *A. biguttula biguttula* exhibiting over 90% complete BUSCOs. Assemblies from *B. tabaci* were significantly extensive and intricate, but *T. palmi* exhibited the highest N50 and average contig lengths, signifying abundant and superior assemblies.

Analyses conducted on selected databases (4IN, SWISS-PROT-Insecta, KONAGAbase) revealed several conserved genes across read length assemblies. Chitin synthase 1, ArgK, STPPs, and RyR were uniformly identified across various read lengths, indicating their independence from sequencing depth. Functional targets, including acetylcholinesterase, C-type lectins, and JNK pathway genes, were identified, demonstrating strong identification despite fluctuations in read depth. Analysis of the meta-database identified 1,909 possible target genes, with 1,302 conserved across all four insect orders. Notable conserved gene families comprised acetylcholine receptors, chitin deacetylases, STPPs, ArgK, and RyR. RyR was identified in all 14 inter-order expression comparisons, supporting its significant evolutionary conservation and functional importance. These targets were consistently found irrespective of the transcriptome construction approach, hence enhancing their suitability as targets for pest management methods.

The consistent identification of targets such as STPP, RyR, ArgK, and chitin deacetylases in both methodologies strengthens their credibility for pest management strategies. Thus, both methodologies yield complementary insights, establishing a more robust and cohesive framework for identifying multi-order conserved gene targets in cotton-infesting insect pests. Table 6 outlines the most significant gene targets identified using both methodologies, emphasizing their critical physiological functions and conservation across insect orders.

**Table 6:** Summary of target identification from two approaches and their conservation across insect orders (Hemiptera, Lepidoptera, Orthoptera, and Thysanoptera).

Gene name	Identified from	Functional role	Conserved across four orders
ArgK	Both approaches	Energy metabolism	Yes
RyR	Both approaches	Calcium signalling	Yes
STPP	Both approaches	Signal transduction	Yes
Acetylcholinesterase	Both approaches	Neural transmission	Yes
Chitin synthase 1	Both approaches	Exoskeleton biosynthesis	Yes

**Fig. 3:**
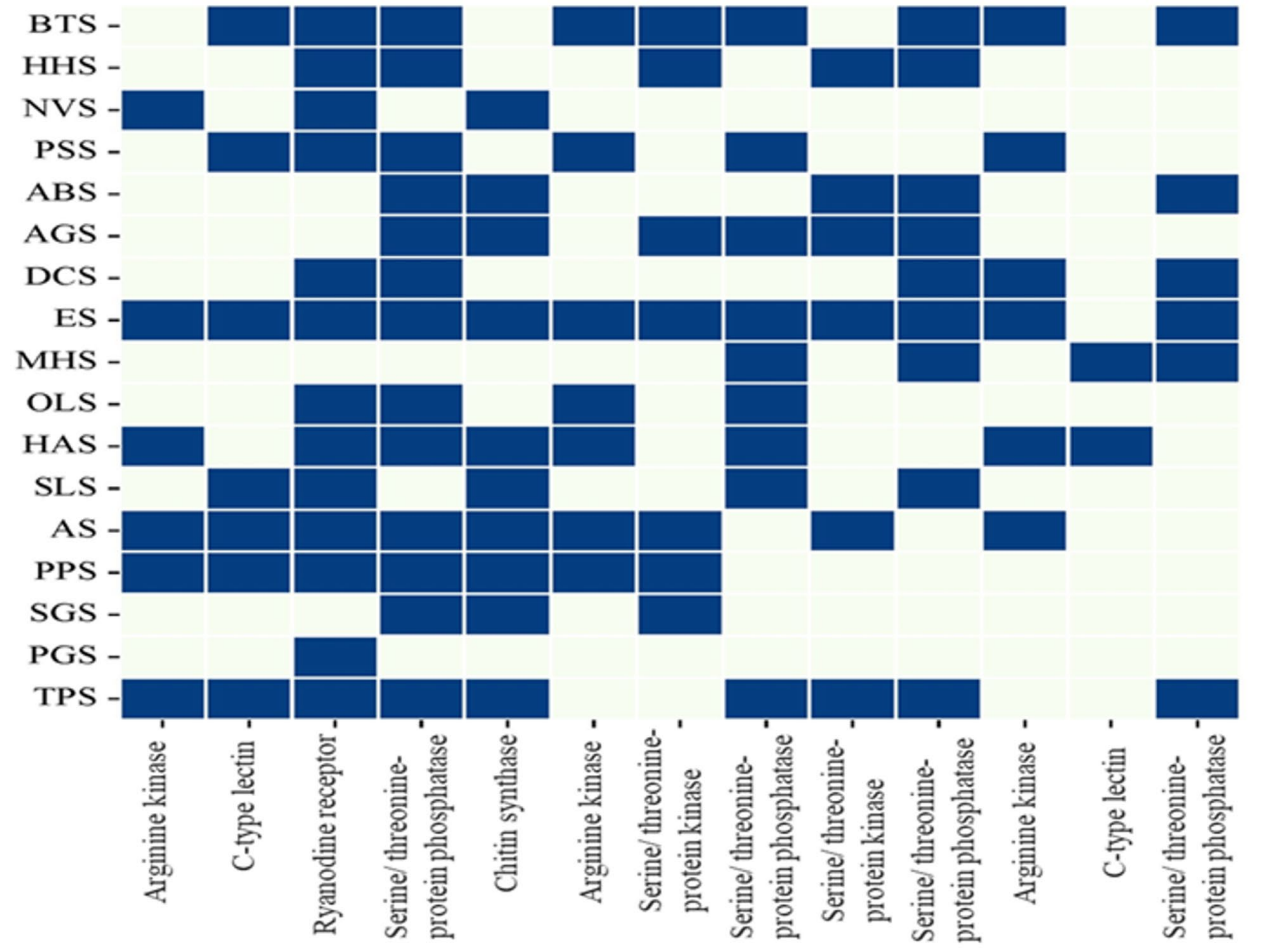
Distribution of key enzymes and receptors across pest species. This heatmap shows the presence of specific enzymes and receptors across various pest species, indicated by blue squares. Each row represents a pest species, while each column represents an enzyme or receptor. The pest species analyzed include *Bemisia tabaci* (BTS), *Halyomorpha halys* (HHS), *Nezara viridula* (NVS), *Phenococcus solenopsis* (PSS), *Amrasca biguttula biguttula* (ABS), *Aphis gossypii* (AGS), *Dysdercus cingulatus* (DCS), *Empoasca onukii* (ES), *Maconellicoccus hirsutus* (MHS), *Oxycarenus laetus* (OLS), *Helicoverpa armigera* (HAS), *Schistocerca gregaria* (SLS), *Agrotis ipsilon* (AS), *Pectinophora gossypiella* (PPS), *Schistocerca gregaria* (SGS), *Poecilocerus pictus* (PGS), and *Thrips palmi* (TPS).

The heatmap (Fig. 3) visually displays the distribution of targets in the specified pest species. For instance, the presence of ArgK in *B. tabaci* (BTS) suggests its involvement in energy metabolism within this species. The significance of chitin synthase in exoskeleton development is shown by its presence in *Dysdercus cingulatus* (DCS) and *Empoasca onukii* (ES). The observed distribution patterns in the heatmap indicate that these receptors play a crucial role in numerous physiological processes in these pests and might be potential targets for efforts aimed at managing pests. This comprehensive visualisation facilitates comprehension of the biochemical variability and functional importance of these proteins among various pest species, offering valuable insights into their contributions to pest biology and prospective strategies for pest management.

### GSEA studies and KEGG pathway enrichment analysis

**Fig. 4:**
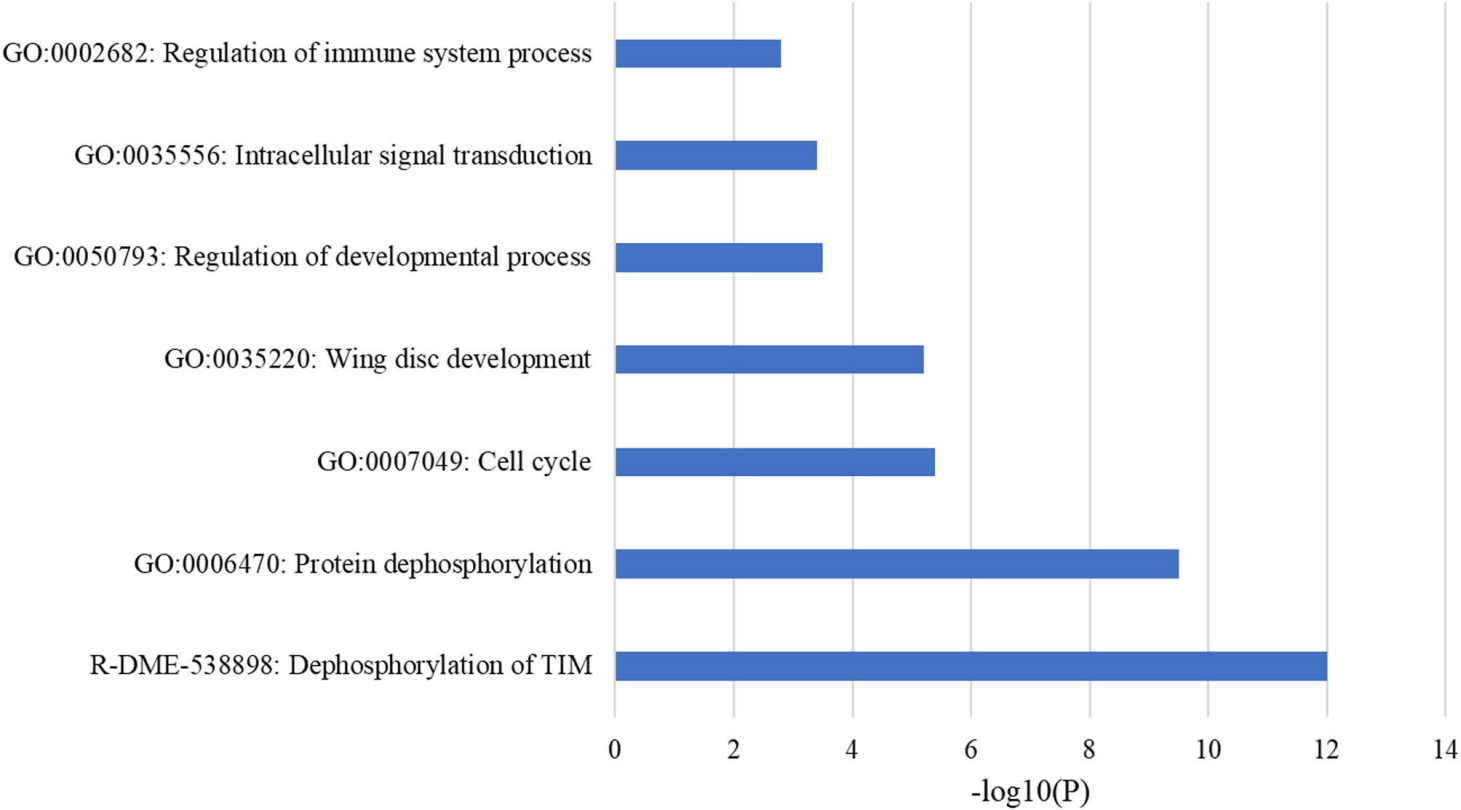
Figure reveals the results for Gene Set Enrichment Analysis (GSEA) performed by MetaScape The bar chart displays the significance of different biological processes and pathways associated with a set of protein targets, as measured by the -log10(P) value. The higher the -log10(P) value, the more statistically significant the association.

To achieve a more profound understanding of the functional implications of the commonly expressed genes across various insect orders, Gene Set Enrichment Analysis (GSEA) was performed utilizing gene lists obtained from SWISS-PROT, KONAGAbase, and InsectBase annotations. The GO enrichment analysis identified multiple biological processes essential to insect physiology, such as protein dephosphorylation, regulation of the cell cycle, development of wing discs, intracellular signal transduction, and modulation of the immune system (Fig. 4). Significantly, the process of "Dephosphorylation of TIM," which plays a crucial role in the regulation of circadian rhythms, was found to be highly enriched, underscoring its vital function in the coordination of behavior and metabolism in insects. The processes are closely connected to developmental transitions, metamorphosis, neural activity, and immune readiness, underscoring the biological significance of the identified genes.

**Fig. 5:**
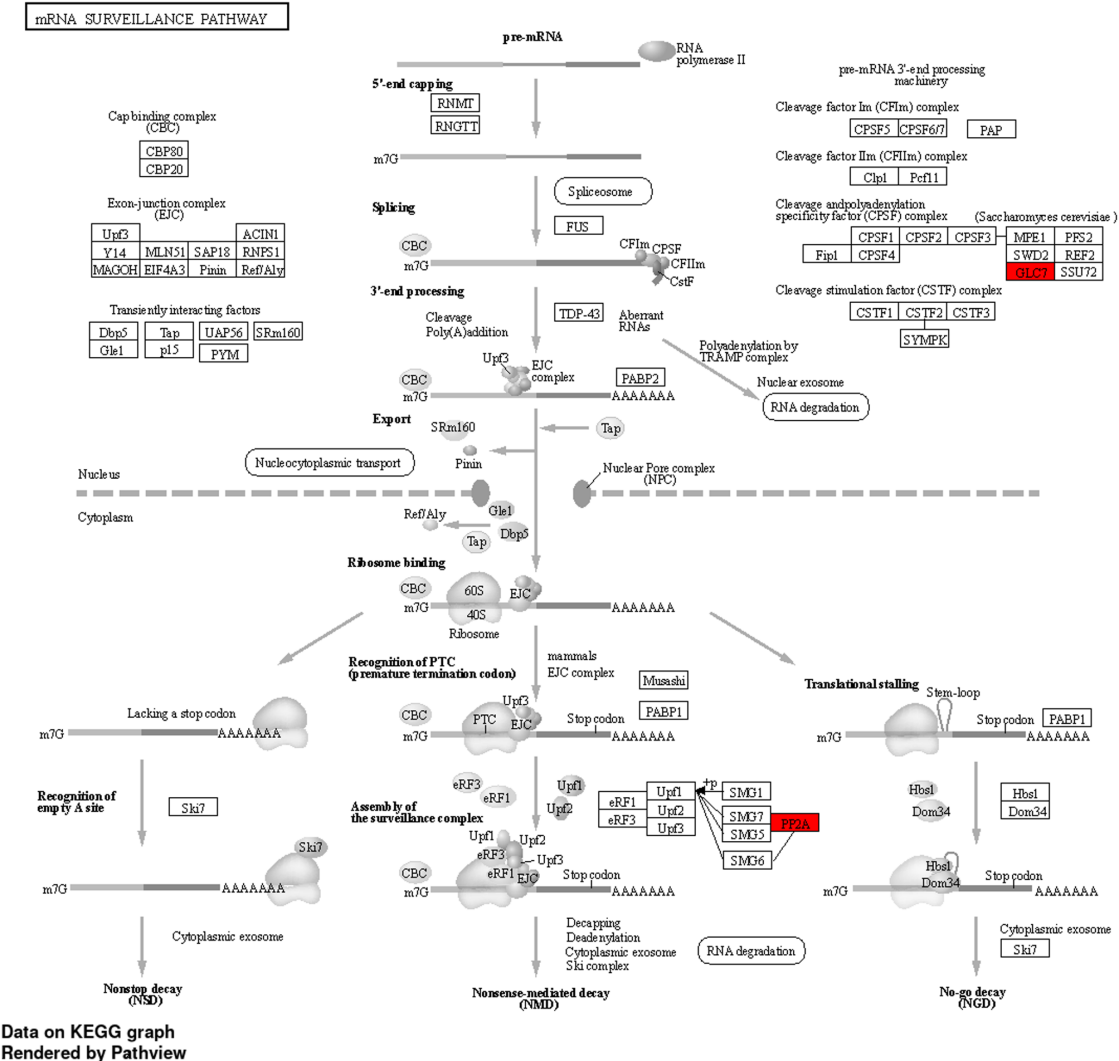
KEGG enrichment analysis performed using PathView which shows PP2A and GLC7 (highlighted in red) enriched in the mRNA surveillance pathway. Pathway image adapted from KEGG (Kanehisa Laboratories, www.kegg.jp/kegg/). Used with permission.

In addition to these findings, KEGG pathway analysis (Fig. 5) revealed significant enrichment in the mRNA surveillance pathway, which serves as a quality control mechanism to maintain the accuracy of gene expression. Multiple essential genes associated with nonsense-mediated decay (NMD), nonstop decay (NSD), and no-go decay (NGD) were identified, highlighting their significance in post-transcriptional regulation. The detection of Protein Phosphatase 2A (PP2A), a member of the STPPs family, is particularly noteworthy, as it plays a critical role in mRNA processing and turnover by regulating phosphorylation dynamics. The presence of PP2A, marked in red in the KEGG diagram, supports the GO result on dephosphorylation and confirms the strength of the combined enrichment strategies. The data collectively highlight that the genes frequently expressed are not just structurally conserved but also play a crucial role in sustaining vital biochemical and physiological processes across various insect species.

### qPCR validation

**Fig. 6:**
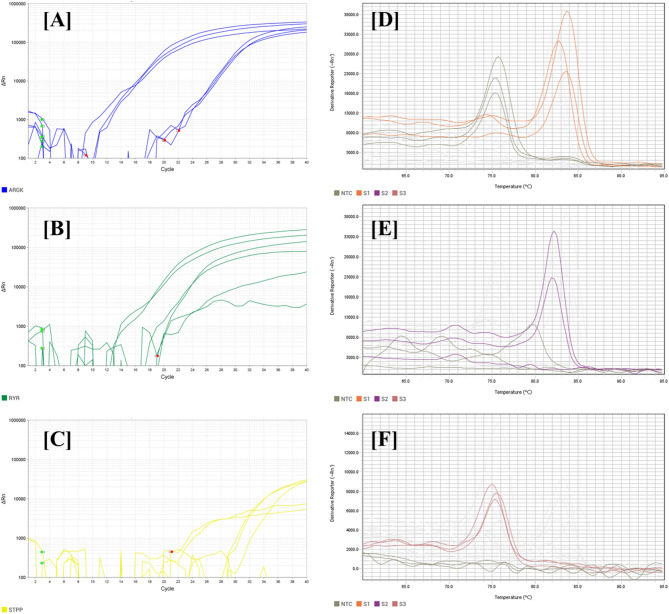
Amplification plots (**A**, **B**, **C**) and melting curves (**D**, **E**, **F**) for ArgK (**A**, **D**), RyR (**B**, **E**) and STPP (**C**, **F**).

The third objective of our investigation was to verify the existence of the identified targets in the model species we chose for our analysis, *Oxycarenus laetus*. Three genes out of ten have been confirmed to exist within the species *O. laetus*. The genes are ArgK, RyR, and STPP. The qPCR protocol outlined in the methodology produced the subsequent amplification and melting plots. All three targets were successfully amplified, as demonstrated by the amplification plots and melt curves obtained in Fig. 6. The specificity of amplification is validated for each target through the observation of a singular melt peak. The Ct values and melt peak heights indicate that ArgK and RyR exhibited superior and more consistent amplification in comparison to STPP. ArgK exhibits significant amplification in S1 with a low mean Ct (23.08), signifying high target abundance. RyR demonstrates significant amplification in S2, with a mean Ct of 22.02, signifying moderate target abundance. STPP demonstrates amplification in S3, although with a high mean Ct value of 32.13.

## Discussion

The current study sought to identify conserved gene targets among economically significant cotton-infesting insect pests from four primary orders: Hemiptera, Lepidoptera, Orthoptera, and Thysanoptera. This was achieved through a dual transcriptome assembly strategy, which included read length-wise assembly (Approach 1) and species-wise assembly (Approach 2). Through the analysis of 104 RNA-Seq datasets spanning 17 pest species, we successfully generated and validated high-quality transcriptomes using bowtie2 alignment and BUSCO completeness assessment. Extended read lengths (PE150) and species-specific assemblies resulted in enhanced assembly continuity and improved gene recovery, especially in *T. palmi, A. biguttula biguttula*, and *B. tabaci*, with the assemblies reaching over 90% BUSCO completeness. The assemblies established a basis for functional gene mining using three curated databases: 4IN, SWISS-PROT (Insecta), and KONAGAbase, along with a comprehensive meta-database. This approach facilitated the identification of a core set of conserved and biologically significant gene targets across the four insect orders.

Among the most consistently identified and conserved targets across both approaches were Arginine kinase (ArgK)^51^, Ryanodine receptor (RyR)^52^, and Serine/Threonine protein phosphatases (STPPs)^53^, each playing a vital role in essential physiological processes. ArgK plays a crucial role in ATP buffering within invertebrate energy metabolism and has been shown to cause RNAi-mediated suppression of larval development^35^ and viability in both *H. armigera* and *A. gossypii*^54^. RyR, a key regulator of intracellular calcium release, is currently targeted by diamide insecticides like chlorantraniliprole^11^ and flubendiamide^55^; however, resistance has developed in *P. xylostella*^56^ and *Spodoptera frugiperda*^57^, highlighting the need for alternative control strategies. STPPs mediate signal termination^58^ via dephosphorylation and are involved in various processes, including gene regulation, cell cycle progression, and responses to stress. Their detection corresponds with enhanced GO terms like “protein dephosphorylation” and "cell cycle regulation," which are further substantiated through GSEA.

The GSEA and KEGG pathway enrichment analyses provided essential functional insights into the transcriptomic findings. The GO enrichment results highlighted processes including the dephosphorylation of TIM, cell cycle progression, and immune system regulation, which are indicative of crucial biochemical processes associated with development, circadian rhythm, and defense mechanisms in insects. The KEGG enrichment analysis revealed that the mRNA surveillance pathway is a significantly pathway. Key regulators such as PP2A^59^, recognized as a specific isoform of STPP, were emphasized for their roles in nonsense-mediated decay (NMD) and post-transcriptional regulation, which are essential for preserving transcript fidelity. The findings confirm that the conserved genes identified are not just sequence matches; they play an active role in essential molecular processes that support insect viability and behavior.

RyRs play a crucial role in muscle function, neural signaling, and developmental coordination by mediating the swift release of calcium from intracellular stores, which is essential for muscle contraction and neuromuscular activity. In contrast to mammals, which have three isoforms of RyR (RyR1, RyR2, RyR3), insects generally encode only one RyR isoform, exhibiting around 47% overall sequence identity with the mammalian RyR2^52^. The transmembrane domain, which plays a crucial role in ion conductance and drug binding, exhibits a high level of conservation (~ 70% similarity), positioning it as a promising and specific target for insecticidal compounds. RyR is expressed widely in insects throughout their developmental stages^60^, present in both the central nervous system (CNS) and striated muscles of larvae and adults. RyRs play a vital role in various physiological processes beyond just movement^61^. They influence wing beat frequency, gut peristalsis, reproductive muscle contractions, and are even involved in visual processing, as calcium transients are closely associated with phototransduction in specific insect taxa^62^. The transcriptomic analysis confirmed the consistent presence and expression of RyR genes in all four studied insect orders: Hemiptera, Lepidoptera, Orthoptera, and Thysanoptera. This extensive conservation highlights its crucial function in insect neuromuscular physiology and developmental homeostasis, reinforcing its value as a target for pest management^55^.

Arginine Kinase (ArgK) was consistently identified as one of the three core gene targets across all four insect orders: Hemiptera, Lepidoptera, Orthoptera, and Thysanoptera through both transcriptome assembly approaches, namely read length-wise and species-wise. Our study identified ArgK across all major databases (SWISS-PROT, KONAGAbase, InsectBase) and noted its significant enrichment in GSEA-derived energy metabolism pathways, underscoring its functional relevance in insect physiology. ArgK, a phosphagen kinase, catalyzes the reversible transfer of phosphate from ATP to arginine, resulting in the formation of phosphor-arginine, which serves as a short-term energy buffer. This buffering system is crucial for facilitating rapid and energetically demanding processes, including muscle contraction, flight, molting, and neuronal signaling, particularly in the context of varying metabolic requirements^63^. Our findings corroborate prior research identifying ArgK as a critical metabolic enzyme across multiple insect species. Insect ArgK is primarily expressed in flight muscles and the nervous system, indicating its crucial function in sustaining ATP levels. The silencing of ArgK in *H. armigera* through RNA interference (RNAi) resulted in developmental arrest^64^, decreased pupation, and increased larval mortality^35^. In *Spodoptera exigua*, the knockdown of ArgK disrupted energy metabolism and affected reproduction and molting, underscoring its pleiotropic effects^65^. The detection of ArgK across all four insect orders in this study indicates that this gene is evolutionarily conserved and functionally essential, positioning it as a suitable candidate for broad-spectrum pest control strategies. The lack of ArgK in vertebrates^51^, which utilize creatine kinase, renders it a highly selective and ecologically safe target for RNA interference or small molecule-based interventions. This study confirms the expression of the target in various pest species and supports its functional significance via enrichment analyses and experimental validation.

Serine/Threonine Protein Phosphatases (STPPs) are essential enzymes that catalyze the dephosphorylation of serine and threonine residues in phosphoproteins, acting antagonistically to kinases. The reversible phosphorylation mechanism serves as a critical regulatory layer in nearly all intracellular signaling pathways, encompassing development, gene expression, metabolism, immune responses, and apoptosis^59^. In insect systems, STPPs play a crucial role in processes including molting, cell cycle progression, signal attenuation in MAPK cascades, and coordination of stress responses. The limited number of STPP catalytic subunits is significantly enhanced through their association with diverse regulatory subunits, which facilitates substrate specificity and spatial regulation across various tissues. Our study consistently identified STPPs across all four insect orders (Hemiptera, Lepidoptera, Orthoptera, and Thysanoptera) using transcriptome assembly approaches, with confirmation from three functional databases: SWISS-PROT, KONAGAbase, and InsectBase. The enrichment of STPP-related genes in Gene Ontology terms such as “protein dephosphorylation” and KEGG pathways like mRNA surveillance (via PP2A, a core STPP isoform) underscores their extensive functional roles. The modulation of ecdysone signaling, neurotransmission, germ cell maturation, and innate immunity by STPPs, specifically Protein Phosphatase 1 (PP1) and Protein Phosphatase 2A (PP2A)^66^. PP2A is involved in regulating cuticle protein synthesis and the deactivation of the ecdysone receptor, thereby influencing the insect molting cycle. Numerous naturally occurring compounds have been recognized as inhibitors of STPP, exhibiting insecticidal potential. Cantharidin, derived from blister beetles (*Mylabris* spp.), and endothall, an herbicide analog, act as inhibitors of PP1 and PP2A, leading to cytoskeletal disruption, cell cycle arrest, and apoptosis in insects and other organisms^53,59^. Due to their pivotal function in regulating critical signaling pathways and their conservation across pest taxa, STPPs represent a promising multi-functional target for insect control.

In addition to the three primary targets, additional genes and gene families were identified, many of which serve as potential candidates for pest control. Those included are chitin synthase 1^67–69^ and chitin deacetylase^70,71^, essential for molting and cuticle formation; the ecdysone receptor^72,73^, crucial for hormonal regulation of development; the vitellogenin receptor^74–78^, significant for female reproduction and fecundity; and C-type lectins^79^, which influence immune responses. Genes associated with juvenile hormone metabolism, Ras-related signaling^80,81^, and odorant receptors^82^ and binding proteins were also significant. These genes collectively affect critical physiological pathways, including development, reproduction, behavior, and immunity, and present a wide array of biocontrol opportunities. Many of these targets are either absent or structurally divergent in non-insect species, such as vertebrates, rendering them particularly suitable for species-specific pest management strategies that pose minimal ecological risk.

The expression of the three core gene targets: RyR, ArgK, and STPP was validated in *O. laetus* through qPCR analysis. *O. laetus* was chosen as the validation model based on several important criteria. This pest is emerging as a significant threat in cotton-growing regions, particularly in the context of changing climatic conditions and intensified agricultural practices. Secondly, *O. laetus* was consistently represented in both the read length-based and species-wise transcriptome assemblies, demonstrating high-quality assembly metrics, transcript completeness, and notable mapping rates. *O. laetus* is a non-model pest with limited genomic resources, making it an ideal candidate for demonstrating the applicability of our pipeline in under-studied, agriculturally significant species. All three genes exhibited distinct, sharp melting peaks, thereby confirming the amplification of specific, non-degenerate products. ArgK displayed a melting temperature of around 85–86 °C, suggesting a clean and uniform amplification, consistent with the high transcript abundance noted in the transcriptomic data. RyR exhibited a comparable specific melt peak in the range of 84–85 °C, with slight variations in peak height, which may indicate differences in amplicon yield across samples. STPP exhibited a distinct melt peak at approximately 83–84 °C, accompanied by a marginally lower peak intensity, indicating a potential decrease in amplification efficiency or expression level in *O. laetus*.

The integration of in silico prediction and wet-lab validation in *O. laetus* illustrates the practical relevance of our findings, especially for pests with limited genomic resources. The three primary targets—ArgK, RyR, and STPP—are essential for energy metabolism, calcium signaling, and post-translational regulation, respectively, and are conserved among insect taxa, while being absent or divergent in vertebrates. This evolutionary divergence presents a significant opportunity for the development of targeted pest control strategies that minimize non-target impacts. Future efforts should prioritize functional RNAi assays, optimization of delivery systems, and cross-species efficacy tests, advancing the development of next-generation, gene-specific biopesticides capable of effectively and sustainably managing pest populations beyond the constraints of conventional insecticides.

## Conclusion

This investigation utilized an extensive, two-way transcriptome analysis to identify conserved molecular targets among four important insect pest orders: Hemiptera, Lepidoptera, Orthoptera, and Thysanoptera those present considerable risks to cotton production. A total of 104 RNA-Seq datasets from 17 pest species were compiled utilizing both read length and species-specific methodologies, leading to the generation of high-quality transcriptomes. The process of functional annotation and gene mining utilizing curated databases such as 4IN, SWISS-PROT, and KONAGAbase, along with a comprehensive meta-database, resulted in the discovery of essential gene targets. Within the set of conserved genes, Arginine kinase (ArgK), Ryanodine receptor (RyR), and Serine/Threonine protein phosphatases (STPPs) were reliably identified across all four insect orders. The analyses conducted indicate that these genes are crucial in the processes of calcium signaling, energy metabolism, and the regulation of phosphorylation. The absence or structural divergence of these targets in vertebrates makes them particularly advantageous for implementing selective pest control strategies that minimize off-target effects. Although RyR, ArgK, and STPP were chosen for targeted validation because of their high conservation and known significance to pest physiology, the larger target set i.e., Ras-like GTPases, ecdysone receptors, lectins, odorant-binding proteins, juvenile hormone metabolism genes, chitin biosynthesis enzymes, and vitellogenin receptors were additionally identified by the approach 2.The experimental validation of ArgK, RyR, and STPP expression was conducted in *Oxycarenus laetus*, a significant Hemipteran pest gaining prominence in agriculture. *O. laetus* was chosen for validation because of its reliable transcriptome quality and its presence in both transcriptome methodologies. The results from qPCR demonstrated specific amplification and consistent expression of these targets, underscoring their significance for comprehensive control interventions. The qPCR analysis revealed specific amplification and consistent expression, underscoring their effectiveness as potential targets. Future efforts ought to include functional assays across a broader range of pest models, in addition to the development of gene-targeted bioinsecticides. The combination of transcriptomics, database mining, and experimental validation underscores the potential of advanced, gene-targeted control strategies like RNAi and the development of biopesticides. This combined and comprehensive strategy may address the shortcomings of traditional approaches, leading to more sustainable and precise pest management strategies.

## Electronic supplementary material

Below is the link to the electronic supplementary material.

Supplementary Material 1

Supplementary Material 2

Supplementary Material 3

Supplementary Material 4

## Data Availability

The datasets used for the current study are publicly available in the SRA database. The accession numbers of the data accessed for this study are as follows: SRR1104130, SRR1930109, SRR1943311, SRR4039449, SRR4039450, SRR5110306, SRR5109958, SRR9141102, SRR9141103, SRR9141104, SRR9141105, SRR6782023, SRR13590441, SRR13590442, SRR13590444, SRR13590443, SRR13328464, SRR13328465, SRR8526511, SRR8526512, SRR7184294, SRR7956289, SRR7961920, SRR8320991, SRR4242253, SRR4242254, SRR4242255, SRR4242256, SRR6315672, SRR6315673, SRR8296279, SRR8296281, SRR8296282, SRR8296285, SRR8296287, SRR8113222, SRR8113223, SRR8113224, SRR8113225, SRR8113226, SRR8113227, SRR9705737, SRR9705738, SRR9705739, SRR9705740, SRR9705741, SRR9705742, SRR10389549, SRR10389550, SRR10389551, SRR10389552, SRR10389553, SRR10389554, SRR12975714, SRR12975715, SRR12975716, SRR12975717, SRR12975718, SRR12975719, SRR7657827, SRR7657828, SRR7657829, SRR7657830, SRR7657831, SRR7657832, SRR5040256, SRR4422921, SRR4436881, SRR4436882, SRR4436883, SRR8932991, SRR8932992, SRR8932993, SRR8932994, SRR8932995, SRR8932996, SRR11747758, SRR11748354, SRR12286913, SRR12286914, SRR12286915, SRR12286916, SRR9308190, SRR9308191, SRR11483256, SRR11483257, SRR11961687, SRR11961690, SRR8755468, SRR8755469, SRR13664481, SRR13664482, SRR13664483, SRR13664484, SRR13664485, SRR13664486, SRR1811919, SRR2230609, SRR11177268, SRR11177269, SRR11177270, SRR11177271, SRR11177272 and SRR11177273. More information about the same is included in the supplementary data.
