# Supplementary figures and images for: Two-way transcriptome approach for the identification of common gene targets across four insect orders and its validation in Oxycarenus laetus
Source: Sci Rep. 2025 Jul 9;15:24721. doi: 10.1038/s41598-025-08880-9 (PMC12241319; doi:10.1038/s41598-025-08880-9)

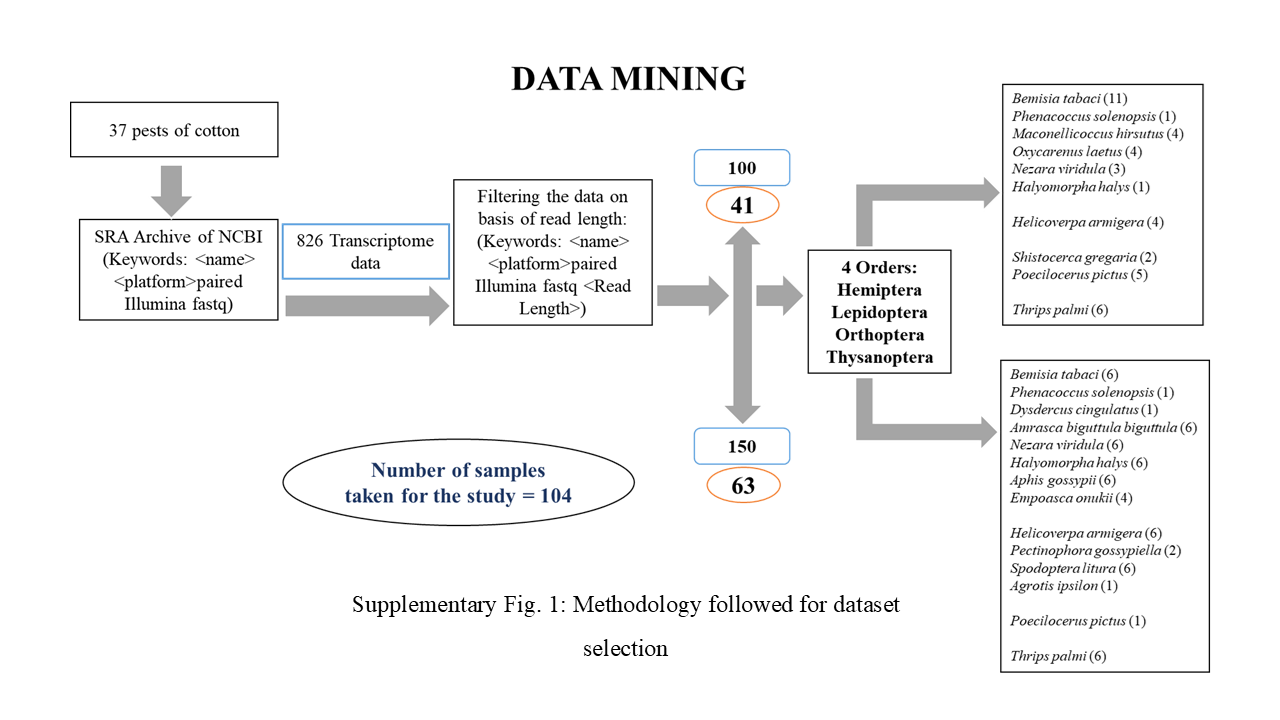

Supplement: Supplementary file 3 — Supplementary Material 3 [file 41598_2025_8880_MOESM3_ESM.tif]
